# Continuous Monitoring of Advanced Hemodynamic Parameters during Hemodialysis Demonstrated Early Variations in Patients Experiencing Intradialytic Hypotension

**DOI:** 10.3390/biomedicines12061177

**Published:** 2024-05-25

**Authors:** Yotam Kolben, Ittamar Gork, David Peled, Shani Amitay, Peleg Moshel, Nir Goldstein, Arik Ben Ishay, Meir Fons, Michael Tabi, Arik Eisenkraft, Yftach Gepner, Dean Nachman

**Affiliations:** 1 Heart Institute, Hadassah Medical Center, Faculty of Medicine, Hebrew University of Jerusalem, Jerusalem 9112001, Israel; yotam@4kosh.com (Y.K.); shani.amitay@mail.huji.ac.il (S.A.); pelegmoshel@gmail.com (P.M.); deannahman@gmail.com (D.N.); 2 Department of Nephrology and Hypertension, Hadassah Medical Center, Faculty of Medicine, Hebrew University of Jerusalem, Jerusalem 9112001, Israel; gorkster@gmail.com; 3 Department of Health Promotion, School of Public Health, Faculty of Medicine and Health, Sylvan Adams Sports Institute, Tel Aviv University, Tel Aviv 6997801, Israel; dpeled94@gmail.com (D.P.); gepner@tauex.tau.ac.il (Y.G.); 4 Biobeat Technologies Ltd., Petah Tikva 4937213, Israel; nirgoldnz@gmail.com (N.G.); arik@bio-beat.com (A.B.I.); meir@bio-beat.com (M.F.); michael@bio-beat.com (M.T.); 5 Institute for Research in Military Medicine, Faculty of Medicine, The Hebrew University of Jerusalem and the Israel Defense Force Medical Corps, Jerusalem 9112102, Israel

**Keywords:** noninvasive blood pressure, end-stage kidney disease, hemodialysis, intradialytic hypotension, remote patient monitoring, photoplethysmography

## Abstract

Intradialytic hypotension (IDH) is a severe complication of hemodialysis (HD) with a significant impact on morbidity and mortality. In this study, we used a wearable device for the continuous monitoring of hemodynamic vitals to detect hemodynamic changes during HD and attempted to identify IDH. End-stage kidney disease patients were continuously monitored 15 min before starting the session and until 15 min after completion of the session, measuring heart rate (HR), noninvasive cuffless systolic and diastolic blood pressure (SBP and DBP), stroke volume (SV), cardiac output (CO), and systemic vascular resistance (SVR). Data were analyzed retrospectively and included comparing BP measured by the wearable devices (recorded continuously every 5 s) and the cuff-based devices. A total of 98 dialysis sessions were included in the final analysis, and IDH was identified in 22 sessions (22.5%). Both SBP and DBP were highly correlated (r > 0.62, *p* < 0.001 for all) between the wearable device and the cuff-based measurements. Based on the continuous monitoring, patients with IDH had earlier and more profound reductions in SBP and DBP during the HD treatment. In addition, nearly all of the advanced vitals differed between groups. Further studies should be conducted in order to fully understand the potential of noninvasive advanced continuous monitoring in the prediction and prevention of IDH events.

## 1. Introduction

End-stage kidney disease (ESKD) patients undergoing maintenance hemodialysis (HD) treatments suffer from various complications during the treatment [1]. For each dialysis session, the patient’s physiological status should be assessed so that the dialysis prescription can be aligned with the goals for the session to achieve the desired rates and total amount of solute and fluid removal, without causing any side effects or complications [2]. Nevertheless, intradialytic hypotension (IDH) is one of the major complications of HD, prevalent in 15–50% of HD sessions, depending on the definition [3]. The Kidney Disease Outcomes Quality Initiative (KDOQI) and the European Best Practice Guidelines define IDH as the presence of a decrease of at least 20 mmHg in systolic blood pressure (BP) or a reduction in mean arterial pressure (MAP) by 10 mmHg, provided that this decrease is associated with clinical events and the need for nursing interventions [4]. Moreover, IDH is associated with increased morbidity and mortality, with growing evidence of repeated cardiac injury during HD sessions [5,6]. Brain and gut ischemia may also occur [7]. It is assumed to result from a reduction in blood volume when the ultrafiltration rate outpaces the vascular refilling rate from the interstitial space [3].

Nevertheless, it has been suggested that IDH may result from hypovolemia, left ventricular diastolic dysfunction, or a "breakdown" in peripheral resistance [8]. BP reduction, in addition to relevant symptoms, indicates that IDH should be suspected. However, since several intrinsic control mechanisms tightly regulate BP in response to ultrafiltration-induced blood volume reduction, periodic blood pressure measurements during HD are of little use in predicting IDH [9]. The invasive Swan-Ganz pulmonary artery catheterization thermodilution technique is considered as the gold standard for diagnosing IDH. Other invasive and noninvasive methods have been proposed for IDH monitoring in the past, with no wide-scale use so far [9,10,11].

**Figure 1:**
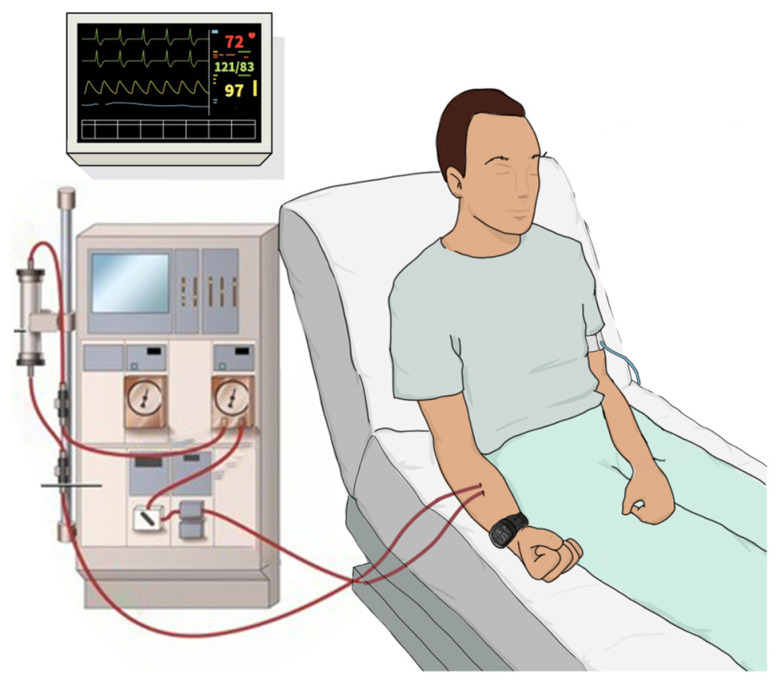
The monitoring sensor is attached to a patient during a dialysis session.

In this study, we continuously monitored patients undergoing HD treatment using a noninvasive, wireless, wearable photoplethysmography (PPG)-based device (BB-613WP, Biobeat Technologies Ltd., Petach Tikva, Israel; Figure 1). The aims of the study were to determine if the PPG-based device can be used to assess BP accurately during HD and to test whether it is possible to identify IDH in the early stage of the HD session. Furthermore, this study aimed to describe the advanced hemodynamic effects of HD, specifically among IDH patients.

## 2. Materials and Methods

### 2.1. Study Design and Overview

This prospective non-interventional study was conducted between 15 January 2021 and 15 February 2022. ESKD patients undergoing chronic intermittent HD treatments in the Hadassah Ein-Kerem medical center were recruited. The study was approved by the Hadassah Ein-Kerem ethics committee (approval number PPGDIA001) and was registered in ClinicalTrials.gov (NCT04680039). Inclusion criteria were patients of both sexes, older than 18 years with ESKD undergoing chronic HD treatments for more than three months, able to adhere to the visit schedule and protocol requirements, and available to complete the study. Exclusion criteria were pregnancy, individuals with a lack of judgment/mental illness, withholding the dialysis session for any reason before initiation, and those employed by the recruiting center. Each participant signed an informed consent form prior to the beginning of the study. Patients were allowed to participate in more than a single HD session. A screening meeting was held up to seven days prior to the first monitored HD session, and participants were monitored during up to 12 dialysis sessions, with the intervention period lasting 90 days at most (see the study flow chart in Supplemental Figure S1). Hemodialysis sessions occurred during the morning, noon, or evening shifts with session lengths being 3–4 h long. Each patient was attended by the same nurse during the whole HD session. Hemodialysis was performed with NIPRO hemodialysis machines and with the use of ELISIO 19H or EISIO 21H dialyzers and solutions with a potassium concentration of 2.0 mmol/L and calcium of 1.5 mmol/L, with some patients requiring adjustments to a potassium concentration of 3.0 mmol/L and/or calcium concentration of 1.25 mmol/L. In each HD session, the PPG-based monitors were attached to the patients’ wrists 15 min before starting the session and until 15 min after completion of the session. The time window before and after each dialysis was used to obtain baseline parameters, to calibrate the PPG device, and to assess the hemodynamic effects after the session termination, respectively. Physiological measurements were recorded continuously every 5 s, and included heart rate (HR), noninvasive cuffless BP, stroke volume (SV), cardiac output (CO), cardiac index (CI), and systemic vascular resistance (SVR). All data were collected and stored during the monitoring period and analyzed retrospectively at the end of the study. At the same time, cuff-based blood pressure measurement devices implemented in the hemodialysis machine were placed on the patients. As the PPG-based device is cuffless, it was placed on the arm with the AV fistula/dialysis venous catheter, so in all cases measurements were taken simultaneously. None of the patients took any medications prior to the beginning of the dialysis session. 

### 2.2. The PPG-Based Monitor

The monitoring devices used in this study (Figure 1) utilize a unique reflective PPG technology, in which part of the transmitted light is reflected from the tissue and detected by a photodiode detector positioned near the light source transmitter. The sensor’s high temporal and quantitative resolution allows it to capture changes in tissue reflectance, from which it derives measurements of several hemodynamic parameters using Pulse Wave Transit Time (PWTT) combined with Pulse Wave Analysis (PWA) [12,13,14,15,16,17]. The device requires a single trimonthly calibration of the HR and BP baseline using an FDA-cleared cuff-based device.

### 2.3. Data Collection and Statistical Analysis

Data were transmitted and stored in real time in a cloud-based web application used by the healthcare providers, with data being coded and fully de-identified. Unusual events were recorded by the researchers. The dataset was segregated into two distinct groups of IDH status: IDH positive and IDH negative based on a 20 mmHg reduction in SBP or 10 mmHg MAP in cuff-based measurement and clinical parameters. Statistical analyses were performed using built-in functions in MATLAB, where numerical data underwent p-value calculation using the t-test and categorical data underwent analysis using the chi-squared test.

Correlation analyses were conducted to explore associations between the PPG-based device and cuff-based measurements for both systolic and diastolic BP with differentiation between IDH positive and IDH negative. Pearson correlation coefficients were computed using MATLAB. Differences between cuff-based measurements and the PPG-based device measurements were computed for instances where both measurements were available simultaneously (IDH negative = 162, IDH positive = 72). These differences were utilized to generate Bland–Altman plots, distinguishing between systolic and diastolic BP, with the level of agreement estimated using 95% confidence intervals.

Dynamic responses of measured parameters from the PPG-based device system were assessed by normalizing the raw data of each treatment to the treatment duration and subsequently averaging across all treatments within each section. The data were then divided into 10 subsections, each representing 10% of the treatment duration. Subsequently, a t-test was employed using MATLAB to compare the responses between IDH-positive and IDH-negative subjects for each subsection.

## 3. Results

In this study, 72 patients were enrolled, among whom 26 patients completed 2 monitored dialysis sessions, resulting in a total of 98 dialysis sessions included in the final analysis. Data were analyzed as per dialysis session, and not per patient. Overall, 22 incidents of IDH occurred. In total, 173,796 measurements of the PPG-based device were collected during an average monitoring period of 3.8 ± 0.3 h per patient (2447 ± 418 measurements per patient). Demographic details are provided in Table 1. There were no adverse events or self-reported discomfort from using the device.

**Figure 2:**
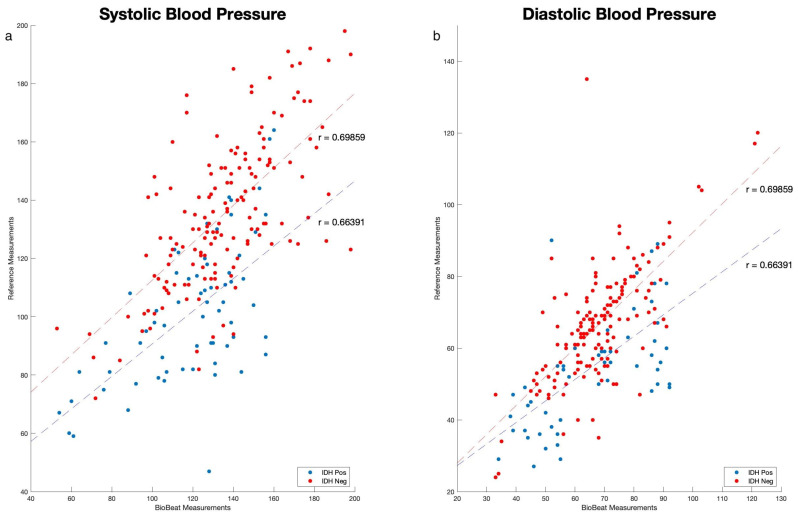
Blood pressure correlation curves between the BP devices: (**a**) systolic blood pressure (SBP) and (**b**) diastolic blood pressure (DBP). Red dots and dashed line are patients that are intradialytic hypotension positive (IDH Pos) and in blue are intradialytic hypotension negative (IDH Neg).

As shown in Figure 2, we performed correlation analyses between point measurements taken by cuff-based BP devices during the dialysis sessions and measurements taken using the PPG-based monitoring devices. High correlations were found in IDH and non-IDH patients for systolic blood pressure (SBP) (r = 0.622 and 0.666, respectively; *p* < 0.001) and diastolic blood pressure (DBP) (0.664 and 0.698, respectively; *p* < 0.001). Values are mean ± SD. 

**Figure 3:**
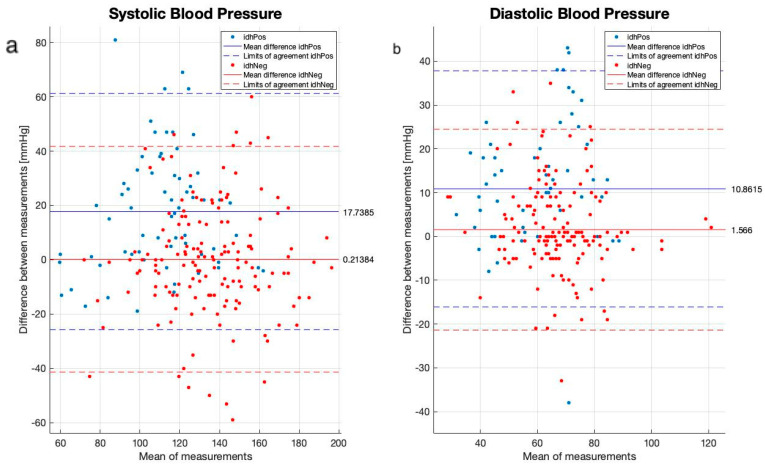
Bland–Altman plots comparing all measurements using the cuff-based and the PPG-based cuffless devices during the dialysis sessions: (**a**) systolic blood pressure; (**b**) diastolic blood pressure. Red dots, IDH positive; blue dots, IDH negative. Dashed line—95% limit of agreement (LOA); continuous line—mean bias.

Next, we compared both the cuff-based and the PPG-based cuffless device using a Bland–Altman analysis (Figure 3). The mean bias of the PPG-based cuffless device was very low for the patients without IDH, with 0.2 mmHg for SBP and 1.5 mmHg for DBP. However, for IDH patients, the PPG-based cuffless device overestimated both SBP and DBP (mean bias of 17.7 and 10.7, respectively), indicating strong specificity but less sensitivity. 

Next, we compared the mean baseline values of the advanced parameters using the PPG-based cuffless device between IDH and non-IDH sessions. No significant differences were found for any of the vitals (Table 2).

**Figure 4:**
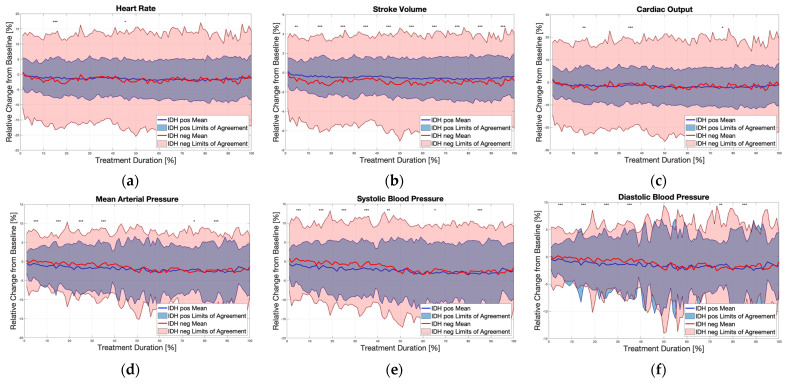

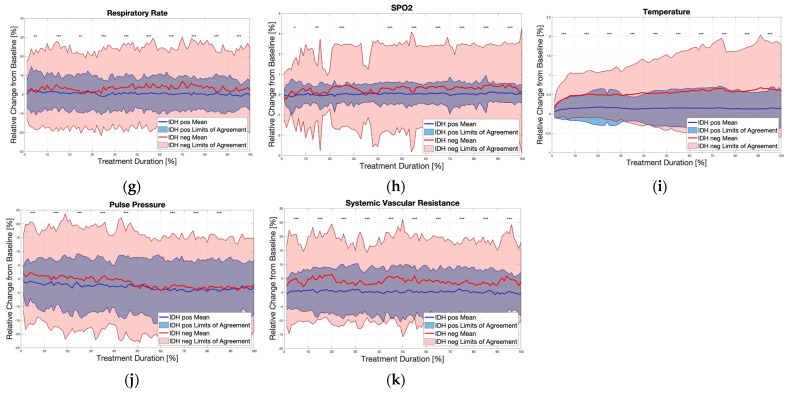
The physiological changes during dialysis in all patients. Baseline measurements were taken as the first 15 min of each dialysis session. Then, changes in each vital were calculated as the relative change during the session (100%) as compared to baseline. A statistical analysis was performed on each 10% separately. An independent sample *t*-test was used to compare the means between IDH and non-IDH for every 10%. The blue (IDH) and the red (non-IDH) lines represent the mean values, and the colored background represent the 95% CI. * *p* < 0.05; ** *p* < 0.01; *** *p* < 0.001.

Changes in the SBP, DBP and advanced measured parameters during the dialysis sessions of all patients are shown in Figure 4. During the HD sessions, patients experiencing IDH exhibited earlier and more pronounced reductions in SBP and DBP compared to those without IDH. Specifically, within the first 10% of the dialysis session, both SBP and DBP significantly decreased in the IDH group (which was clinically defined later by the cuff-based measurements) as compared to the non-IDH group. The analysis of the advanced hemodynamic parameters measured by the wearable device revealed significant differences between the IDH and non-IDH sessions. Specifically, SV, CO, SVR, body temperature, SPO_2_, RR, PP, and MAP were markedly different between the two groups. 

However, there were no significant differences between the groups in terms of risk factors or other hemodynamic parameters identified 15 min prior to the HD session that could predict IDH.

## 4. Discussion

In this preliminary study, we have shown that the wearable remote patient monitoring platform can track hemodynamic parameters during dialysis sessions. 

Previous studies emphasized that the pathogenesis of IDH is still not completely understood. Yet, it is regarded as multifactorial and is the result of a combination of a decline in blood volume and impaired vascular resistance at a background of a reduced cardiovascular reserve, in a way that excessive ultrafiltration may decrease the cardiac output. This is especially true when compensatory mechanisms such as HR, myocardial contractility, vascular tone, and splanchnic flow shifts fail to be optimally recruited [18,19,20,21,22,23]. When diving into the basic characteristics of the patients, an interesting image appears. Many IDH patients also had peripheral vascular disease; this may reflect the vascular contribution to IDH pathogenesis but may also be explained by the technical difficulty in assessing blood pressure in those patients using standard cuff-based measurements. Interestingly, pulmonary hypertension was less common in the IDH group. This finding was not described previously, and its mechanism is obscure. Diuretics were associated with higher rates of IDH. This observation may be explained by intravascular depletion prior to the dialysis, or with the direct vasodilative effect of certain diuretics.

The correlation of the BP measurements between the PPG device and the cuff in both groups is lower than what is accepted in various ISO standards. However, the ISO standard testing is conducted under “sterile” conditions, not allowing the person—among other criteria—to smoke or drink caffeine in the 30 min before BP is taken; they should sit still at least 5 min before measurements are taken, and during measurements the cuffed arm should be kept on a flat surface at the heart level, sitting upright, back straight, feet flat on the floor and not crossed, without movement and no talking. Moreover, this should not be conducted under dynamic circumstances such as hemodialysis. For this reason, we regard the correlation values as good, given the fact they were obtained in a “real-life” scenario that incorporates hemodynamic changes resulting from the shift of fluids. The Bland–Altman analysis revealed a bias in the measurements in the IDH group, but not in the non-IDH group, suggesting suboptimal sensitivity. We could not explain these phenomena, but hemodynamic instability in IDH patients may play a role. Importantly, previous validation studies performed following the ISO standard definitions and under more sterile conditions have shown high correlations between the PPG-based monitor and other devices [24,25].

The early and profound BP reduction in the IDH group is noted at the beginning of the dialysis sessions. Although no hemodynamic parameters helped us significantly predict the IDH events, we assume that a larger cohort may help us with this prediction in the future.

In hypovolemic states, as the CO is reduced due to reduced effective intravascular volume, the main compensatory mechanisms to retain BP are vasoconstriction and SVR elevation [26]. The non-IDH group demonstrated a “classic” reaction to hypovolemia; a reduction in SV and an elevation of SVR. However, the IDH group showed no mean change in SV or SVR. This finding supports the theories that IDH is not a pure hemodynamic response to hypovolemia but a neurohormonal-mediated response, such as a reduction in the sympathetic tone or loss of response to vasopressin [23].

On a general note, as the trend for home dialysis increases, it is important to acknowledge that implementing home dialysis presents several challenges, though the benefits can outweigh these obstacles for many patients [27]. Some challenges are as follows: training requirements of both patients and their caregivers (including learning how to operate the dialysis machine, handle supplies, monitor vital signs, and respond to emergencies); understanding how to troubleshoot common issues that may arise during treatment (e.g., alarms or blood flow problems); adequate space is needed to set up and store equipment and supplies; a reliable source of clean water is required for preparing dialysis fluid; patients performing dialysis at home are at risk of infection, particularly if proper hygiene practices are not followed; social support is often required by patients undergoing home dialysis from family members or caregivers, which may place a strain on relationships and caregiving resources; though home dialysis can be cost effective compared to in-center treatments in the long run, initial setup costs can be significant, and patients must consider the financial implications of ongoing supplies, equipment maintenance, and potential modifications to their home; dialysis can have a significant psychological impact on patients and their families, including feelings of anxiety, isolation, or depression; and patients and caregivers must be prepared to handle emergencies that may arise during dialysis treatment, such as sudden drops in blood pressure or equipment malfunction. This requires access to emergency support services and the ability to respond quickly and effectively. Despite these challenges, it seems that home dialysis offers many benefits, including greater flexibility, improved quality of life, and the ability to perform treatments in the comfort of one’s own home. With proper support, education, and resources, many patients successfully manage home dialysis and experience better health outcomes.

Remote patient monitoring (RPM) can significantly enhance the management of home dialysis by providing real-time and continuous monitoring of relevant patients’ vital signs. This enables the early detection of potential complications or changes in health status, allowing for timely interventions and potentially improved patient outcomes [28]. Moreover, RPM can track patients’ adherence to their dialysis treatment regimen by monitoring treatment sessions, including the duration and frequency of dialysis sessions. Healthcare providers can identify patients who may be struggling with adherence and provide additional support or education as needed. Another potential advantage would be using the valuable data generated by RPM systems in data-driven decision support. Healthcare providers can analyze these data to identify trends, assess treatment efficacy, and make informed decisions to optimize patient care. Overall, it seems as if RPM could play an important role in enhancing the safety, effectiveness, and convenience of home dialysis by providing continuous support, proactive management, and timely interventions to optimize patient outcomes. 

It seems that seeking to identify the optimal intradialytic BP range is vital in driving targeted interventions that clinicians may be able to implement to minimize IDH events and prevent the long-term sequela of IDH. This could include pre-session changes of the dialysis machine setting, adjusting the dialysis settings during the session, and even stopping the session if required. Further investigation should be undertaken to create tools that predict IDH events easily. This, in turn, could allow the early institution of adjustments to the dialysis protocol, preventing IDH from appearing and hopefully avoiding immediate adverse events as well as any long-term sequela. 

Regarding this gap in management, the noninvasive system used in this study can serve as an advanced platform and, when integrated with dialysis sessions, provide an early awareness of the risk of IDH. The continuous monitoring may alert the clinician early regarding hemodynamic changes that may represent certain conditions other than IDH, such as acute cardiac events, sepsis, and infusion reaction. Moreover, it might facilitate efforts of moving dialysis sessions into the home, assuring proper hemodynamic monitoring with early notifications and alerts.

Patients were highly compliant with the use of the device, emphasizing the ease of use when compared with cuff-based devices, yet this setting was relatively short in nature. We have previously shown that patients are highly compliant and satisfied with using the device over a longer period, including 24 h and beyond [19,29].

The limitations of this study include its single-center design, which may restrict the generalization of the findings to other populations or settings. Additionally, this study did not specifically address the impact of ethnicity on the detection and management of IDH, which could limit the applicability of the results to different ethnic groups. There may also be unmeasured confounders such as comorbidities, medication use, or other patient-specific factors that could have influenced the results but were not accounted for in the analysis. Though intervention in the case of intradialytic hypotension is highly important, the focus of this study was on the diagnosis and prevention of IDH, and the response of the dialysis unit team was not documented. This should also be looked at in future studies.

Furthermore, the study was limited by its relatively small number of patients. However, even in this small group, the advantages of using such a platform in the early detection of IDH were apparent. Future studies should aim to include larger patient populations to further evaluate the clinical outcomes associated with the use of the wearable platform. Lastly, we did not collect data on the specific timing of intradialytic hypotension occurrences during the dialysis sessions. Therefore, we were unable to analyze changes in cardiac output, pulse pressure, or stroke volume in relation to the exact timing of IDH events. This should be also looked at in future studies using similar monitoring platforms.

To conclude, this preliminary observational study shows that further studies with larger numbers of participants are required in order to determine whether continuous monitoring using PPG-based devices could serve for the early prediction and prevention of IDH. Interestingly, we show that the pathophysiology of IDH is not explained solely by hypovolemia, but rather another factor that causes a reduction in SVR may play a role.

## Figures and Tables

**Table 1:** Basic characteristics of hemodialysis patients. IDH—intradialytic hypotension, ACEi—Angiotensin-Converting Enzyme inhibitors; ARB—Angiotensin Receptor Blockers; MRA—Mineralocorticoid Receptor Antagonist; CCB—Calcium Channel Blockers.

	non-IDH (n = 76)	IDH (n = 22)	*p*-Value
Age (years)	65.5 ± 9.41	67.0 ± 13.7	0.13
Female (%)	36.4	32.9	0.76
Medications			
Diuretics (%)	18.2	44.7	0.026
ACEi/ARB/MRA (%)	18.2	23.7	0.022
CCB (%)	40.9	35.5	0.028
β-Blockers (%)	40.9	51.3	0.59
α-Blockers (%)	13.6	34.2	0.64
Medical Background			
Heart failure (%)	36.4	39.5	0.39
Hypertension (%)	95.5	72.4	0.066
Diabetes mellitus (%)	77.3	50.0	0.79
Peripheral vascular disease (%)	59.1	15.8	<0.001
Pulmonary hypertension (%)	45.5	21.1	0.021

**Table 2:** Basic mean results of advanced continuous hemodynamic parameters.

	non-IDH (n = 76)	IDH (n = 22)	*p*-Value
Pre-dialysis weight (kg)	96.0 ± 8.02	75.3 ± 12.6	<0.001
Post-dialysis weight (kg)	93.3 ± 7.78	73.7 ± 12.6	<0.001
Weight difference (kg)	−1.71 ± 0.91	−2.12 ± 0.70	0.059
Dialysis duration (hours)	4.10 ± 0.35	3.85 ± 0.31	0.004
Heart rate (beats/min)	87 ± 12	83 ± 14	0.18
Oxygen saturation (%)	98.0 ± 0.7	97.7 ± 1.9	0.59
Respiratory rate (breaths/min)	12 ± 1	12 ± 2	0.39
Temperature (°C)	36.0 ± 0.1	36.0 ± 0.2	0.12
Cardiac Output (L/min)	7.37 ± 1.1	7.22 ± 1.7	0.41
Stroke volume (mL)	85.1 ± 7.8	86.4 ± 9.2	0.56
Systolic blood pressure (mmHg)	123 ± 34	136 ± 32	0.20
Diastolic blood pressure (mmHg)	64 ± 19	67 ± 16	0.34
Mean arterial pressure (mmHg)	84 ± 23	90 ± 20	0.27
Pulse pressure (mmHg)	59 ± 22	69 ± 23	0.20
Systemic vascular resistance (dyn·s·cm^−5^)	947 ± 340	1040 ± 283	0.30

## Data Availability

The data presented in this study are available on request from the corresponding author. The data are not publicly available due to privacy and ethical definitions of the Hadassah Medical Center.

## Supplementary Materials

The following supporting information can be downloaded at: https://www.mdpi.com/article/10.3390/biomedicines12061177/s1, Figure S1: The study flow chart.
