# Supplementary figures and images for: Continuous Monitoring of Advanced Hemodynamic Parameters during Hemodialysis Demonstrated Early Variations in Patients Experiencing Intradialytic Hypotension
Source: Biomedicines. 2024 May 25;12(6):1177. doi: 10.3390/biomedicines12061177 (PMC11200556; doi:10.3390/biomedicines12061177)

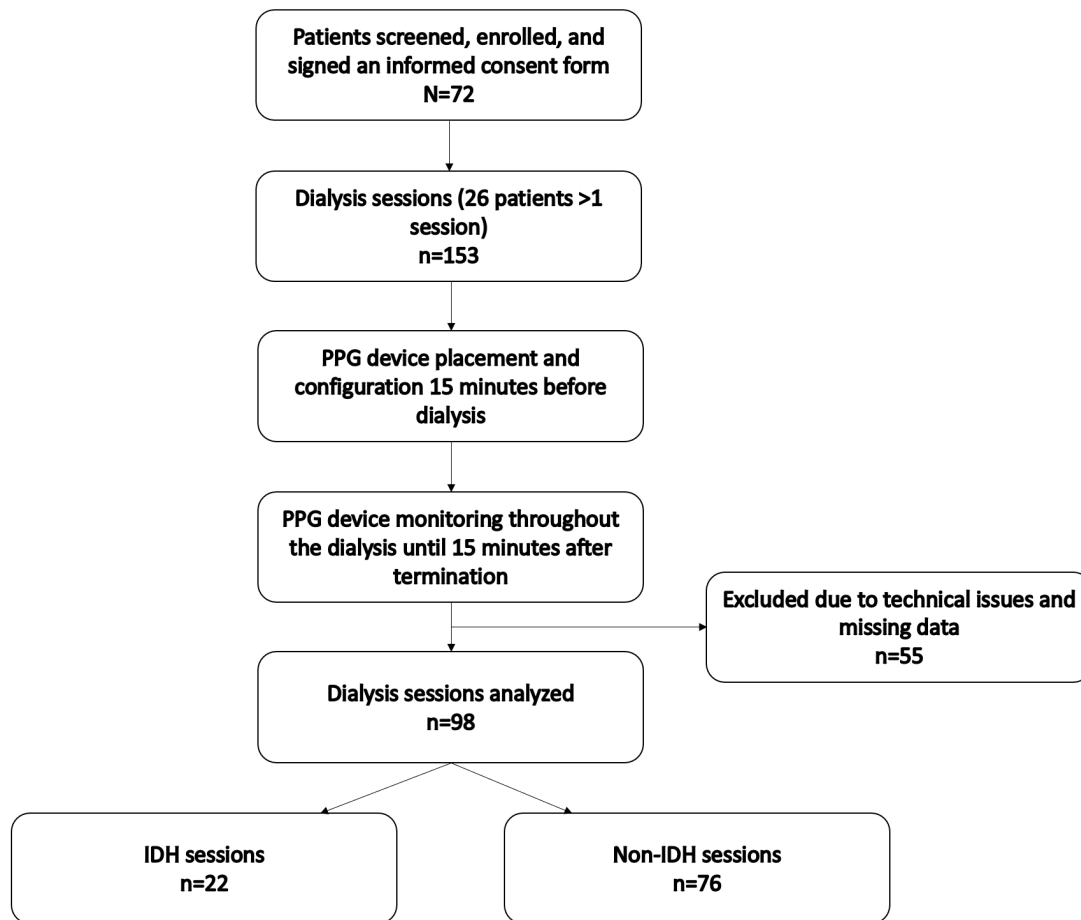

Figure S1: The study flow chart.

Supplement: Supplementary file 1 [file biomedicines-12-01177-s001.zip › biomedicines-2973080-supplementary.pdf]
